# Chemically modified non-coding RNAs in cancer

**DOI:** 10.1017/erm.2025.10007

**Published:** 2025-06-09

**Authors:** Lulu Yang, Boyang Wang, Zhaohui Gong

**Affiliations:** 1 Department of Biochemistry and Molecular Biology and Zhejiang Key Laboratory of Pathophysiology, School of Basic Medical Sciences, Health Science Center, Ningbo University, Ningbo, Zhejiang, China; 2 Department of Thoracic Surgery, The First Affiliated Hospital of Ningbo University, Ningbo, Zhejiang, China

**Keywords:** cancer, chemical modification, diagnosis, non-coding RNA, therapy

## Abstract

**Background:**

Non-coding RNAs (ncRNAs) are transcribed RNA molecules that do not encode proteins but regulate diverse biological processes. Dysregulation of ncRNAs is implicated in cancer, where chemical modifications such as N6-methyladenosine (m6A), N4-acetylcytidine (ac4C), and glycosylation critically influence their function. However, these modifications, as precise regulators of ncRNA activity, have been less well-documented and understood in tumorigenesis and cancer progression.

**Methods:**

This article systematically analyzes the roles of chemically modified ncRNAs – ribosomal RNA (rRNA), circular RNA (circRNA) and others – in cancer biology, synthesizingevidence from published studies on their mechanistic involvement in malignancy.

**Results:**

We reveal how specific chemical modifications drive oncogenesis, impact cancer diagnosis, and affect therapeutic responses, while also exploring their prognostic potential. Furthermore, we highlight emerging connections between ncRNA epitranscriptomics and cancer.

**Conclusions:**

This review provides novel insights into ncRNA epitranscriptomics as emerging biomarkers and intervention targets for precision oncology.

## Introduction

Non-coding RNAs (ncRNAs) are functional RNA molecules genomically transcribed and historically undervalued due to lack of potential for protein or peptide translation. They regulate messenger RNA (mRNA) stability/translation, RNA processing and modification, protein transport, and chromosome structure. Functionally they are classified as housekeeping ncRNAs (e.g., transfer RNA (tRNA), ribosomal RNA (rRNA), small nuclear RNA (snRNA), small nucleolar RNA (snoRNA)), and regulatory ones (e.g., long non-coding RNA (lncRNA), Piwi-interacting RNA (piRNA), microRNA (miRNA), small interfering RNA (siRNA), and circular RNA (circRNA)) (Ref. 1).

RNA chemical modifications, crucial for determining biological polymer functionality, expand the diversity of RNA through post-transcriptional modifications (PTMs). It has uncovered over 170 distinct types of chemical modifications present in RNA molecules (Refs 2, 3). These alterations influence RNA stability, distribution and activity, and are linked to diseases including cancer. As a complex genetic disorder resulting from the accumulation of mutations, cancer is characterized by dysregulated uncontrolled cell proliferation and gene expression. The cancer burden in China is increasing to over 4 million new cases and approximately 3 million deaths (Ref. 4). ncRNAs play pivotal roles in cancer pathogenesis (Ref. 5), and contribute to cancer progression by modulating gene expression via RNA modifications (Ref. 6). A comprehensive exploration of the roles and significance of ncRNA chemical modifications and associated signaling pathways in cancer holds promise for advancing cancer treatment strategies.

## ncRNA and cancer

ncRNAs are significantly involved in either facilitating or inhibiting cancer progression across a wide range of cancer types (Ref. 7). The roles of several common types of ncRNAs in cancer are summarized in Table 1.

**Table 1.** Common types of ncRNAs and their roles in cancer

ncRNAs			Cancer	Role in cancer	Cancer-related mechanisms and/or functions of ncRNAs	Reference
Housekeeping ncRNAs	snoRNA	SNORD50A/B	BC	Tumor suppressor	Inhibits tumor growth via suppressing the binding of FTase to K-Ras	(Ref. 10)
		SNORD17	HCC	Oncogene	Promotes cell proliferation, cell cycle progression and inhibits apoptosis	(Ref. 12)
		SNORA23	PDAC	Oncogene	Promotes cell proliferation and invasion via upregulating SYNE2	(Ref. 13)
		SNORD113–1	HCC	Tumor suppressor	Inhibits tumorigenesis via phosphorylation of ERK1/2 and SMAD2/3	(Ref. 11)
	rRNA	/	CRC	Oncogene	Promotes ribosome assembly via hCINAP and tumor proliferation	(Ref. 14)
Regulatory ncRNAs	miRNA	miR15/16	CLL	Tumor suppressor	miR15/16 genes are deleted or down-regulated	(Ref. 17)
		miR–31	EC	Oncogene	Promotes cell proliferation and invasion via inhibiting Hippo pathway; predicts poor prognosis	(Ref. 18)
		miR–34	CRC	Tumor suppressor	Inhibits tumorigenesis via losing miR–34 and activating the IL6-STAT3 pathway	(Ref. 20)
	lncRNA	PCA3	PC	Oncogene	Promotes cell proliferation and invasion through AR signaling pathway	(Ref. 28)
		PD-L1-lnc	LUAD	Oncogene	Promotes cell proliferation and invasion; inhibits apoptosis	(Ref. 29)
		lncRNA BC069792	BC	Tumor suppressor	Inhibits cell proliferation and migration	(Ref. 31)
		KIMAT1	NSCLC	Oncogene	Promotes cell proliferation and invasion	(Ref. 30)
	siRNA	/	/	Tumor suppressor	Inhibits tumor progression	(Ref. 36)
	piRNA	piRNA–14633	CC	Oncogene	Promotes cell proliferation, migration, and invasion via the METTL14/CYP1B1 signaling axis	(Ref. 39)
		piR–36712	BC	Tumor suppressor	Inhibits cell proliferation, invasion and migration via binding with SEPW1P RNA	(Ref. 41)
		piR–823	MM	Oncogene	Maintains stemness of MM cells by upregulating DNMT3B	(Ref. 38)
		piR–39980	Fibrosarcoma	Tumor suppressor	Inhibits cell proliferation via interacting with RRM2	(Ref. 40)
	circRNA	hsa_circ_0003258	PC	Oncogene	Promotes cell migration and EMT	(Ref. 45)
		circSATB2	NSCLC	Oncogene	Promotes cell proliferation, migration, and invasion	(Ref. 46)

BC: breast cancer; CC: cervical cancer; CLL: chronic lymphocytic leukemia; CRC: colorectal cancer; EC: endometrial carcinoma; HCC: hepatocellular carcinoma; LUAD: lung adenocarcinoma; MM: multiple myeloma; NSCLC: non-small cell lung cancer; PC: prostate cancer; PDAC: pancreatic ductal carcinoma.

### snoRNA in cancer

snoRNAs, small nucleolar ncRNAs (60–300 nucleotides), are abundant in eukaryotic nucleoli (Ref. 8). They play dual roles in cancer progression (Ref. 9). Tumor-suppressive snoRNAs like SNORD50A/B has been shown to inhibit BS (BC) by binding K-RAS (Ref. 10). Similarly, SNORD113–1 inhibits tumorigenesis by regulating MAPK/ERK and TGF-β signaling pathways (Ref. 11). Conversely, oncogenic snoRNAs such as SNORD17 promote hepatocellular carcinoma (HCC) through a positive feedback loop involving nucleolar phosphoprotein 1 (NPM1) and human Myb-binding protein 1A (MYBBP1A) upon p53 inactivation (Ref. 12), and SNORA23 promotes tumor cell proliferation and invasion by upregulating the expression of spectrin repeat containing nuclear envelope 2 (SYNE2) (Ref. 13). Consequently, snoRNAs are implicated in tumorigenesis and may emerge as pivotal biomarkers for diagnosing and predicting the outcome of various cancers.

### rRNA in cancer

rRNAs affect cancer progression through gene expression regulation (Ref. 6), ribosome assembly modulation (Ref. 14), and oncogenic protein translation (driving proliferation/transformation). Their roles as therapeutic targets and prognostic markers (Ref. 15), highlight novel strategies for cancer diagnosis, treatment, and outcome prediction, offering innovative insights into cancer management.

### miRNA in cancer

miRNAs, short RNAs (19–25 nucleotides), regulate gene expression and exhibit dual roles in cancer as oncogenes or tumor suppressors (Ref. 16). A foundational study revealed reduced miR-15/miR-16 expression in chronic lymphocytic leukemia (CLL) (Ref. 17), while miR-31 promotes endometrial cancer (EC) by inhibition of the Hippo pathway (Ref. 18). The miR-34 family, regulated by tumor suppressor p53, controls cell growth, apoptosis, cell cycle (Ref. 19). Notably, miR-34a loss has been shown to promote colorectal cancer (CRC) and predict poor survival in CRC patients via activating the IL6-STAT3 signaling pathway (Ref. 20). Importantly, miR-34a may serve as the most promising miRNA drugs for cancer treatment (Ref. 21), underscoring diagnostic and therapeutic value of miRNAs in cancer management. Hence, miRNAs broadly influence cancer progression by modulating cell growth, invasion/metastasis, angiogenesis, and cellular transformation (Refs 5, 22).

### lncRNA in cancer

lncRNAs, transcripts exceeding 200 nucleotides (Ref. 23), regulate cancer development by modulating proliferation, differentiation, and metastasis (Refs 24, 25 26, 27). Oncogenic lncRNAs include prostate cancer antigen 3 (PCA3), elevated in prostate cancer (PCa) patients’ urine as an early diagnostic biomarker (Ref. 28), and lncRNA programmed cell death ligand 1 (PD-L1), which accelerates lung adenocarcinoma (LUAD) by enhancing c-Myc transcriptional activity (Ref. 29). Furthermore, lncRNA KIMAT1 may represent a therapeutic target for KRAS-driven lung cancer (LC) (Ref. 30). Conversely, tumor-suppressive lncRNAs like BC069792 inhibit BC by sponging miR-658 and miR-4739 as a competitive endogenous RNA (ceRNA), upregulating KCNQ4, and suppressing AKT phosphorylation to block metastasis (Ref. 31). These findings underscore the pivotal function of lncRNAs in gene regulatory networks, indicating their promise as trustworthy diagnostic indicators or targets for cancer treatment (Ref. 32).

### siRNA in cancer

siRNAs regulate eukaryotic genome expression and function by modulating endogenous genes and protecting the genome against invading nucleic acids (Ref. 33). Their primary function is in RNA interference (RNAi), a highly specific regulation that governs gene expression in a base-pairing manner (Ref. 34). They modulate tumor-related signaling pathways (Refs 34, 35), and offer targeted cancer therapy potential by silencing oncogenes with low doses, minimal side effects, highlighting their promise in precision oncology (Ref. 36).

### piRNA in cancer

piRNAs (24–31 nucleotides) exert regulatory effects by interacting with Piwi proteins (Ref. 37). Oncogenic piR-823 enhances DNA methylation in multiple myeloma (MM) (Ref. 38), while piRNA-14633 drives cervical cancer (CC) via the methyltransferase-like protein 14 (METTL14)/CYP1B1 signaling axis (Ref. 39). Tumor-suppressive piR-39980 targets ribonucleotide reductase subunit M2 (RRM2) (Ref. 40), and piR-36712 downregulation results in elevated selenoprotein W pseudogene 1 (SEPW1), which may inhibit p53, upregulating Slug to promote cell proliferation, invasion, and migration (Ref. 41). Their dual roles, mediated through upstream events (e.g., methylation, gene silencing), underscore their impact on tumorigenesis. Their involvement in critical regulatory networks positions piRNAs as promising biomarkers for early cancer detection and therapeutic targets, offering the potential for precision interventions in oncology.

### circRNA in cancer

circRNAs, covalently closed loops formed via precursor mRNA (pre-mRNA) back-splicing (Ref. 42), influence cancer progression by modulating metastasis and invasiveness (Refs 43, 44). For instance, hsa_circ_0003258 promotes PCa metastasis by upregulating Rho GTPase-activating protein 5 (ARHGAP5) expression, stabilizing histone deacetylase 4 (HDAC4) mRNA via insulin-like growth factor 2 mRNA-binding protein 3 (IGF2BP3) binding, activating of the ERK signaling pathway, and triggering epithelial-mesenchymal transition (EMT) (Ref. 45). Similarly, circSATB2 drives non-small cell lung cancer (NSCLC) progression via the miR-326/FSCN1 axis and facilitates exosome-mediated intercellular communication, highlighting its diagnostic potential (Ref. 46). Their aberrant expression across various cancers positions them as promising biomarkers for early detection and therapeutic targets (Refs 47, 48).

## Chemical modifications in ncRNAs

RNA chemical modifications predominantly occur in ncRNAs, regulating gene expression. Aberrant modifications are linked to disease etiology, with distinct modifications diversely influencing RNA metabolism and function, underscoring their critical regulatory roles.

### N6-methyladenosine (m6A)

**Figure 1.**
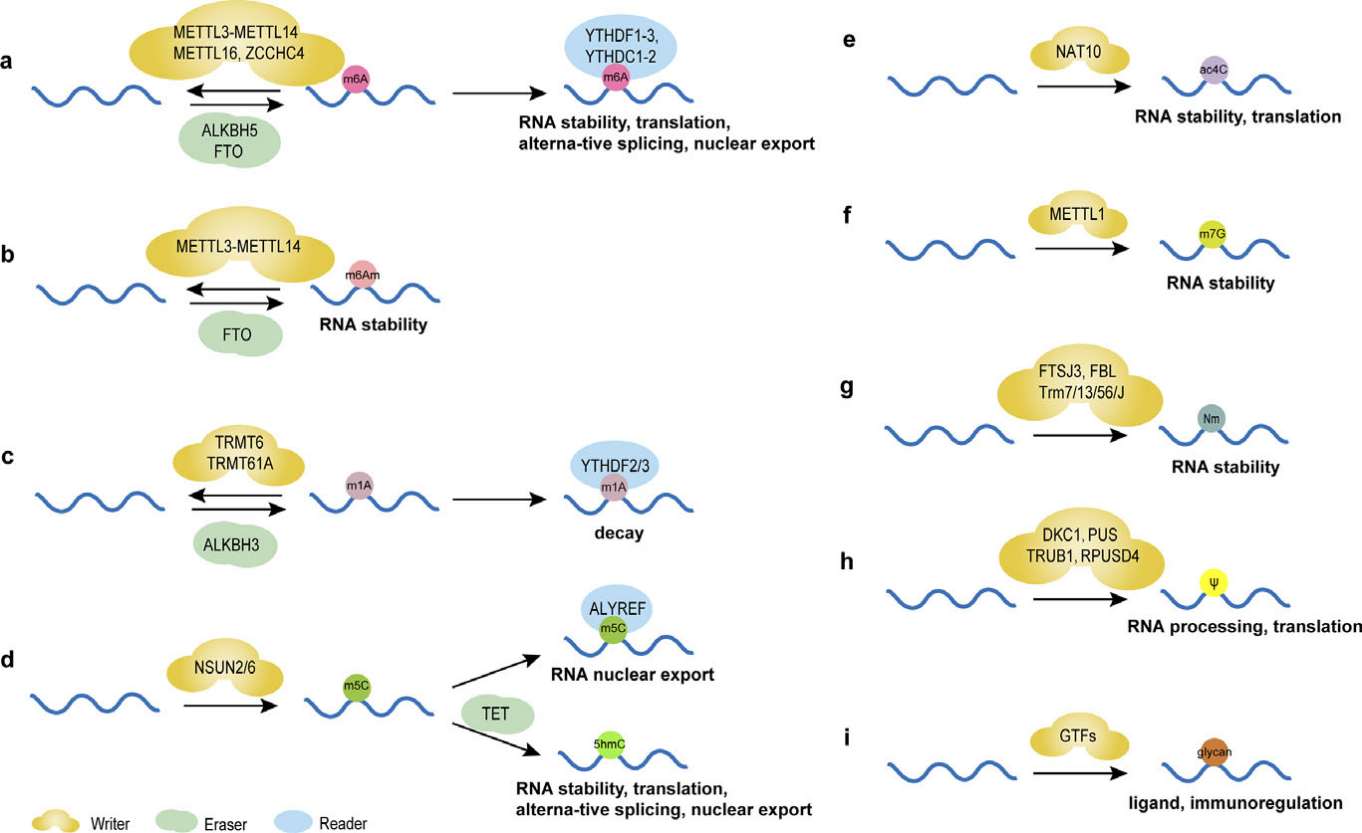
Chemical modifications of RNAs and their main functions. (**A**) m6A modification regulates the stability, translation, alternative splicing and nuclear export of RNAs mediated by writers, including METTL3-methyltransferase-like 14 (METTL14), methyltransferase-like 16 (METTL16), Zinc Finger CCHC-Type Containing 4 (ZCCHC4), erasers FTO and α-ketoglutarate-dependent dioxygenasehuman AlkB homolog 5 (ALKBH5), and reader proteins YTH structural domain family proteins 1–3 (YTHDF1–3) and YTH structural domain-containing proteins 1–2 (YTHDC1–2). (**B**) m6Am modification regulates the stability of RNAs mediated by writers, including METTL3-METTL14 and erasers FTO. (**C**) m1A modification regulates the decay of RNAs mediated by writers, including tRNA methyltransferase 6 (TRMT6) and tRNA methyltransferase 61A (TRMT61A), erasers ALKBH3 and reader proteins YTHDF2/3. (**D**) m5C modification regulates the nuclear export of RNAs mediated by writers NOL1/NOP2/SUN domain family member (NSUN) 2/6 and reader proteins Aly/REF export factor (ALYREF). 5hmC is formed from 5mC by oxidation of 10–11 translocation (TET) proteins, regulating the stability, translation, alternative splicing and nuclear export of RNAs. (**E**) ac4C modification regulates the stability and translation of RNAs mediated by writers N-acetyltransferase 10 (NAT10). (**F**) m7G modification regulates the stability of RNAs mediated by writers methyltransferase-like protein-1 (METTL1). (**G**) Nm regulates the stability of RNAs mediated by writers, including FtsJ homolog 3 (FTSJ3), rRNA 2’-O-methyltransferase fibrillarin (FBL) and tRNA methyltransferase (Trm) 7/13/56/J. (**H**) Ψ modification regulates the processing and translation of RNAs mediated by writers, including DKC1, PUS, probable tRNA pseudouridine synthase 1 (TRUB1) and RNA pseudouridylate synthase domain containing 4 (RPUSD4). (**I**) Glycosylated RNAs act as ligands in immunoregulation under the regulation of glycosltransferases (GTFs).

N6-methyladenosine (m6A), the most prevalent and distinctive form of RNA methylation modification (Ref. 6), involves reversible methyl group addition by ‘Writers’ (methyltransferases), removal by ‘Erasers’ (demethylases), and recognition by ‘Readers’ (m6A-binding proteins) (Figure 1a), critically regulating RNA metabolism and function (Refs 49, 50). This dynamic modification influences gene expression in physiological and pathological conditions, including cancer (Ref. 51). For instance, methyltransferase-like protein 3 (METTL3)- mediated m6A facilitates miRNA maturation by guiding DiGeorge syndrome critical region 8 (DGCR8) to primary miRNA (pri-miRNA) (Ref. 52). Cigarette smoke condensate (CSC) may be a promoter of pri-miR-25 maturation through METTL3-mediated m6A modification, activating the AKT-p70S6K signaling pathway and potentially promoting cancer development (Ref. 53). m6A also drives circRNA biogenesis and alters lncRNA stability (Ref. 7). However, m6A dysregulation may disrupt ncRNA stability, localization, and function, thereby affecting the regulation of gene expression and promoting the proliferation, invasion and metastasis of tumor cells (Ref. 54). Collectively, m6A epitranscriptomic regulation bridges RNA modification with disease etiology, highlighting its potential as a biomarker and intervention target.

### N6,2’-O-dimethyladenosine (m6Am)

m6Am, the methylation of adenosine’s nitrogen at position 6 (Ref. 55), modulates the stability of ncRNAs (Figure 1b) (Ref. 49), thereby influencing disease development (Ref. 56). Though research is limited, further studies may reveal novel therapeutic avenues, particularly in metabolic and immunotherapeutic applications.

### N1-methyladenosine (m1A)

m1A, the N1-methylation of adenine, influences immune responses by promoting T-cell expansion through tRNA modification (Figure 1c) (Ref. 57). Detected via methylated RNA immunoprecipitation sequencing (MeRIP-seq) (Ref. 58), advancements in high-throughput sequencing now enable precise localization and quantification of m1A modifications, revealing their roles in ncRNAs (Refs 58-60). Further research could deepen understanding of its biological functions, offering insights into ncRNA mechanisms and therapeutic potential.

### 5-methylcytosine (m5C)

m5C, the methylation at the fifth carbon of cytosine, represents a crucial RNA epigenetic modification regulating gene expression by influencing RNA stability and nuclear export (Figure 1d) (Ref. 61). In tRNAs, m5C enhances translation efficiency through optimized codon-anticodon pairing on tRNAs and mRNAs (Ref. 62). Moreover, m5C is essential for cell functions like stress responses and metabolic processes (Ref. 63). m5C alterations in ncRNAs can affect tumor progression (Refs 62, 63, 64), suggesting their potential clinical value.

### N4-acetylcytidine (ac4C)

ac4C, the acetylation of cytosine’s fourth nitrogen, ensures translation accuracy and was initially identified in tRNAs (Refs 65, 66). It also exists in rRNAs and mRNAs, affecting RNA stability and function (Figure 1e) (Refs 67, 68, 69). Linked to diseases like cancer, ac4C abnormalities in ncRNAs highlight their potential as diagnostic biomarkers and therapeutic targets.

### N7-methylguanosine (m7G)

m7G, the methylation at guanosine’s 7th nitrogen, occurs in RNAs like mRNA and tRNA, regulating post-transcriptional processing, stability, translation, degradation, and interactions with RNA-binding proteins (RBPs) (Figure 1f) (Refs 70, 71, 72, 73). Additionally, tools like m7GDisAI have been established to identify potential disease-related m7G loci (Ref. 74), aiding research into its roles in RNA biology and potential therapeutic applications.

### 2’-O-methylation (2’-O-Me, Nm)

Nm modification involves methylating RNA ribose’s 2′-hydroxyl group, stabilizing piRNAs, maintaining tRNA function, protecting mRNAs from decapping exoribonuclease (DXO) degradation, and ensuring rRNA biogenesis(Figure 1g) (Ref. 75). Like m6A, Nm modification is increasingly studied for its diverse functions and regulatory mechanisms in ncRNAs (Ref. 76), underscoring its biological importance and potential as a therapeutic target.

### Pseudouridine (Ψ)

Pseudouridine (Ψ), termed the “fifth nucleoside” of RNA, is formed by pseudouridine synthases (PUSs) through a β-glycosidic bond linking uracil’s C-5 to ribose’s C-1, creating a structural isomer of uridine (Refs 77, 78). Abundant in ncRNAs like tRNAs and snRNAs, Ψ modifications are involved in cellular activities and contribute to pathological conditions (Refs 79, 80). For example, PUS10 regulates nuclear miRNA processing and cytoplasmic tRNA pseudouridylation (Ref. 79), while PUS7 overexpression correlates with poor prognosis in patients with glioblastoma (GBM) by enhancing tyrosine kinase 2 (TYK2) translation efficiency of via tRNA pseudouridylation (Figure 1h), thereby promoting glioblastoma stem cell (GSC) growth (Ref. 81). The dyskerin pseudouridine synthase 1 (DKC1) gene encodes a dyskerin protein with PUS activity, which binds to and catalyzes the uridine isomerization of target RNAs to Ψ. Cancer progression and poor prognosis are linked to the overexpression of DKC1, which has been detected in a range of cancer types (Ref. 6, 82). However, the specific function of Ψ-modified ncRNAs in cancer biology is yet to be fully understood.

### Glycosylation

Glycosylation, the enzymatic addition of sugar residues to proteins or lipids in the endoplasmic reticulum and Golgi apparatus, is closely linked to cancer progression by influencing tumor growth, invasiveness, and immune evasion (Figure 1i) (Ref. 83). Traditionally associated with proteins and lipids, this paradigm was challenged when Flynn et al. discovered glycosylated small non-coding RNAs (sncRNAs) produced via the classic protein N-glycosylation pathway. These glycosylated sncRNAs, found on cell surfaces, interact with Siglec receptors to regulate immune responses (Ref. 84). Glycosylation also modifies Ψ in tRNA anticodons, critical for post-embryonic growth by maintaining codon translation and protein stability (Ref. 85). *In situ* imaging reveals dynamic glycosylated RNA levels during disease and physiological processes: they increase during pro-inflammatory monocyte/macrophage-vascular endothelial cell interactions and decrease during immune differentiation and BC metastasis (Ref. 86). Cell-surface glycosylated RNAs are recognized by endothelial P-selectin, implicating them in neutrophil-mediated inflammation and tumor development (Ref. 87). These findings underscore glycosylation’s expanded role beyond classical substrates, highlighting its regulatory functions in RNA biology, immune modulation, and disease mechanisms. Glycosylated RNAs may serve as novel biomarkers or therapeutic targets in cancer and inflammatory disorders, though further research is needed to unravel their precise molecular roles and clinical potential.

## The role of chemically modified ncRNAs in cancer

Cancer incidence, notably lung, colorectal, and liver cancers, has risen in China with high mortality rates. RNA chemical modifications exert a great influence on cancer (Table 2), with aberrant RNA modifications potentially promoting cancer cell growth and self-renewal. Targeting these RNA chemical alterations may offer novel strategies for cancer treatment.

**Table 2.** Chemically modified ncRNAs in cancer

RNA modifications	Cancer	ncRNAs	Regulators	Role	References
Ψ	GBM	tRNA	PUS7	Regulates the TYK2-STAT1 pathway, promotes cell growth, and predicts poor prognosis	(Ref. 81)
	HCC	18S rRNA	RAS	SNORA24 mediates modifications that affect translation accuracy	(Ref. 105)
	CRC	28S rRNA	GCLC	SNORA56 mediates modifications that inhibit ferroptosis, and promote cell proliferation	(Ref. 100)
	BC	r RNA	DKC1	Being associated with tumorigenesis, affects the function of ribosome and the synthesis of protein	(Ref. 120)
Nm	NSCLC	28S rRNA	SCD1	SNORD88C mediates modifications that promote cell proliferation, invasive metastasis and inhibit autophagy	(Ref. 96)
	CRC	r RNA	NOP58	ZFAS1 targets and recruits NOP58, while SNORD12C and SNORD78 mediate modifications that promote cell proliferation, migration and inhibit apoptosis	(Ref. 97)
m7G	LUAD	tRNA	METTL1, WDR4	Promotes cell proliferation, migration, invasion,and predicts poor prognosis	(Ref. 95)
	LC	let–7e	METTL1	Inhibits cell migration	(Ref. 70)
m6A	PDAC	seRNA	CFL1, METTL3, YTHDC2, MLL1	Enhances chromatin accessibility and promotes oncogene transcription	(Ref. 88)
	LUAD	LCAT3	METTL3, FUBP1	Promotes cell proliferation, migration and invasion and is associated with poor prognosis	(Ref. 89)
	HCC	circGPR137B	FTO	Inhibits cell proliferation, invasion, hepatocellular carcinoma infiltration and lung metastasis	(Ref. 90)
	PDAC	miR–25–3p	METTL3	Activate PHLPP2-AKT pathway to promote cell migration and invasion	(Ref. 53)
	CRC	lncRNA XIST	METTL14, WTAP, YTHDF2	Inhibits cell proliferation, migration and invasion	(Ref. 91)
	CRC	lncRNA RP11	METTL3	Promotes cell migration, invasion and EMT	(Ref. 102)
	CRC	circNSUN2	YTHDC1	Associated with poor prognosis and promotes liver metastasis	(Ref. 103)
	CRC	circ1662	METTL3	Promotes CRC cell invasion and migration by accelerating nuclear translocation of YAP1	(Ref. 101)
m5C	GC	NR_033928	NSUN2, GLS	Promotes cell growth; inhibits apoptosis; predicts poor prognosis	(Ref. 94)
	CC	circE7	METTL3	Being associated with polysome and inhibits cell growth	(Ref. 43)
	HCC	PVT1	NOP2	Promotes carcinogenesis, cell proliferation and stem cell-like properties	(Ref. 92)
	ESCC	NMR	NSUN2, BPTF	Promotes the metastasis and invasion of ESCC and enhances the resistance to cisplatin	(Ref. 93)

Abbreviations: BC: breast cancer; CC: cervical cancer; CLL: chronic lymphocytic leukemia; CRC: colorectal cancer; EC: endometrial carcinoma; ESCC: esophageal squamous cell carcinoma; GBM: glioblastoma; GC: gastric cancer; HCC: hepatocellular carcinoma; LC: lung cancer; LUAD: lung adenocarcinoma; NSCLC: non-small cell lung cancer; PC: prostate cancer; PDAC: pancreatic ductal carcinoma.

### Chemically modified ncRNAs in cancer occurrence and development

RNA modifications play critical roles in the development of cancer through diverse mechanisms. For instance, Li et al. highlighted the impact of m6A modification in super-enhancer RNA (seRNA) on histone modification and oncogene expression in pancreatic ductal adenocarcinoma (PDAC) (Ref. 88). Additionally, the upregulation of lncRNA LCAT3, facilitated by m6A modification, promotes the growth, migration as well as invasiveness of LC cell via LCAT3-FUBP1-cMYC axis, leading to a poor prognosis (Ref. 89). Conversely, circGPR137B is identified as a cytoplasmic sponge for miR-4739, which in turn upregulates fat mass and obesity-associated protein (FTO)’ expression. The demethylation of circGPR137B by FTO, which targets m6A, has been found to inhibit cell growth, thereby suppressing HCC metastasis (Ref. 90). METTL3 hypomethylation, induced by CSC via the transcription factor NFIC, enhances m6A methylation of pri-miR-25, accelerating its maturation via NF-κB activating protein (NKAP)-Drosha-DGCR8 complexes to activate oncogenic AKT signaling in the initiation and progression of PDAC (53). Yang et al. also found that METTL14, by forming a complex with Wilms’ tumor 1-associated protein (WTAP), mediates m6A modification of lncRNA XIST, leading to its degradation by YTH structural domain family proteins 2 (YTHDF2) and subsequently inhibiting CRC proliferation and metastasis (Ref. 91). Moreover, m5C methyltransferases drive oncogenesis by catalyzing m5C modifications of target RNAs (Refs 92, 93). In gastric cancer (GC), m5C modification of lncRNA NR_033928 is associated with its upregulation in cells and tissues, affecting cell growth and apoptosis (Ref. 94). The specific disruption of circE7 in CC cells has been demonstrated to lead to decreased E7 protein levels and inhibited cancer cell growth (Ref. 43). The deletion of methyltransferase-like protein-1 (METTL1) and WD repeat domain 4 protein (WDR4) impairs m7G modification of tRNA, reducing the growth, colony formation, and invasiveness of LC cells (Ref. 95). Additionally, METTL1 was found to influence the stability and maturation of let-7e by m7G modification at the G11 site, thereby inhibiting LC progression (Ref. 70). SNORD88C was identified as an oncogenic snoRNA that mediates Nm modification of 28S rRNA, affecting the translation of stearoyl-CoA desaturase1 (SCD1) and inhibiting cellular autophagy, thereby promoting the metastasis of NSCLC (Ref. 96). Similarly, lncRNA ZFAS1 recruits NOP58 to mediate SNORD12C/78-dependent Nm modification, stabilizing rRNA and regulating downstream genes to control cancer cell proliferation and apoptosis (Ref. 97). These findings underscore RNA modifications as central regulators of oncogenic pathways, offering potential therapeutic targets. The interplay between RNA modifications, non-coding RNAs, and protein complexes highlights their multifaceted roles in cancer biology, emphasizing the need for further research to translate these insights into clinical strategies.

### Chemically modified ncRNAs in cancer diagnosis and prognosis

RNA modifications offer promising avenues for addressing limitations in current cancer biomarkers, which often lack specificity and sensitivity (Refs 98, 99). In CRC, SNORA56-driven pseudouridylation (Ψ) of 28S rRNA promotes cell proliferation and correlates with poorer 5-year survival, suggesting it as a prognostic biomarker (Ref. 100). Similarly, circ1662 (Ref. 101) and m6A-modified lncRNA RP11 (Ref. 102) – upregulated by zinc-finger E-box binding protein 1 (ZEB1) – emerge as diagnostic and prognostic markers for CRC metastasis, while m6A-altered circNSUN2 is linked to liver metastasis (Ref. 103). In HCC, m6A-modified miRNAs show superior diagnostic accuracy over traditional biomarkers like AFP for early detection (104), and SNORA24-directed Ψ modifications influence translational fidelity (Ref. 105), suggesting utility in predicting therapeutic responses. Furthermore, a comprehensive analysis of m6A-associated lncRNAs in HCC has shed light on their potential mechanisms in regulating the immune microenvironment, offering new insights into their role and prognostic value in the disease (Ref. 106). In the context of ovarian cancer (OC), the expression of ALKBH5 has been associated with resistance to platinum-based chemotherapy (Ref. 107), suggesting that m6A modification and its regulators may serve as potential biomarkers for the diagnosis of cancer. Current studies emphasize RNA modifications’ dual roles as disease drivers and biomarkers, urging further research to standardize detection methods. Integrating these modifications into existing diagnostic frameworks could enhance early detection, and improve outcomes across diverse cancers.

### Chemically modified ncRNAs in cancer therapy

**Figure 2.**
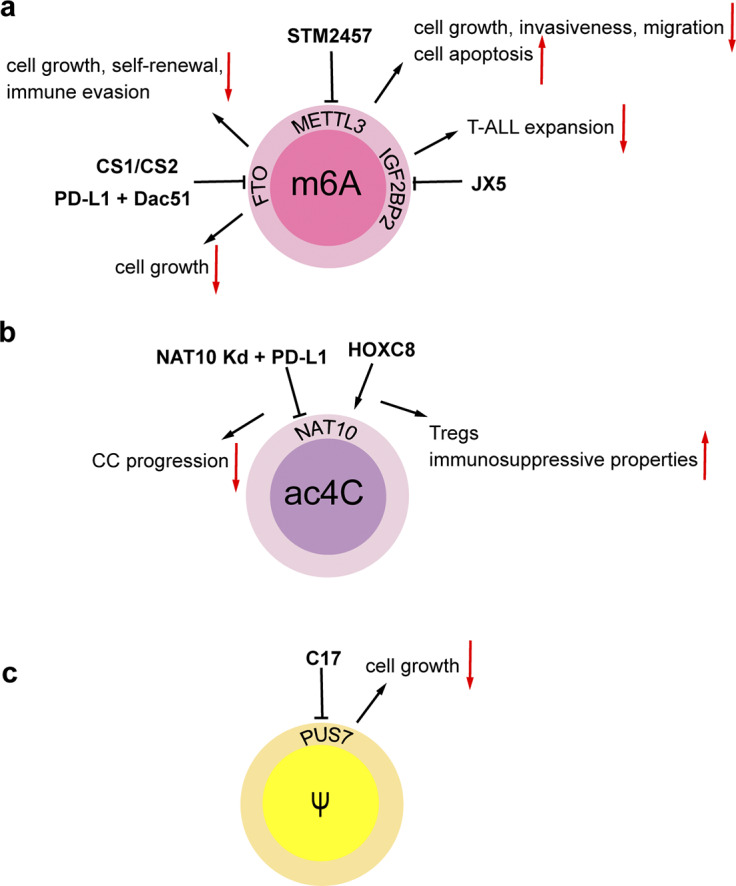
Regulatory agents in chemically modified RNAs. (**A**) The METTL3 inhibitor STM2457 inhibits cell growth, invasiveness, migration, and enhances cell apoptosis in intrahepatic cholangiocarcinoma (ICC). The FTO inhibitors CS1 and CS2 attenuate leukemia stem/initiating cell growth, self-renewal and immune evasion in multiple types of cancers. Combination of PD-L1 blockade and the FTO inhibitor Dac51 inhibits cell growth in in melanoma and lung cancer. The IGF2BP2 inhibitor JX5 suppresses the expansion of T-cell acute lymphoblastic leukemia (T-ALL). (**B**) HOXC8 activates NAT10 and induces the ac4C modification of FOXP1 mRNA, thereby enhancing the immunosuppressive properties of tumor-infiltrating regulatory T cells (Tregs). NAT10 knockdown contributes to the effectiveness of PD-L1 blockade efficacy, thereby suppressing cervical cancer (CC) progression. (**C**) The PUS7 inhibitor C17 inhibits cell growth and tumor progression in glioblastoma.

Aberrant RNA modifications are emerging as promising therapeutic targets in cancer, with inhibitors and immunotherapies showing preclinical efficacy (Refs 108, 109, 110). The METTL3 inhibitor STM2457 suppresses growth, invasiveness, and migration of intrahepatic cholangiocarcinoma (ICC) cells, induces apoptosis, and triggers cell cycle arrest, thereby significantly suppressing ICC progression and exhibiting superior anti-tumor effects (Ref. 111). FTO has been identified as a significant contributor to cancer cell growth and evasion of immune responses (Refs 112, 113, 114). The utilization of small molecule FTO inhibitors, such as CS1 and CS2, has shown promising anti-cancer properties by directly interacting with FTO’s catalytic site. These inhibitors effectively suppress the demethylating function of FTO, hinder its binding to target mRNAs, and show significant inhibitory effects on various cancers like breast and pancreatic cancers (Ref. 115). Moreover, FTO promotes tumor cell glycolytic metabolism through epitranscriptomic regulation, leading to T-cell suppression and induced tumor immune evasion. The combination of PD-L1 blockade and the FTO inhibitor Dac51 has demonstrated enhanced tumor growth inhibition and accelerated activation of CD8^+^ T cells, resulting in improved tumor cell eradication (Ref. 112). Additionally, in T-cell acute lymphoblastic leukemia (T-ALL), the IGF2BP2 inhibitor JX5 disrupts NOTCH1 receptor’s mRNA stability, overcoming chemoresistance (Ref. 116). Furthermore, the overexpression of YTH structural domain family proteins 1 (YTHDF1) in CRC has been correlated with CRC metastasis, implying that YTHDF1-m6A-ARHGEF2 may be a promising target for therapeutic intervention (Ref. 117). Studies have also highlighted the role of ac4C modification by N-acetyltransferase 10 (NAT10) in promoting the development of CC, with immunotherapy targeting NAT10 showing a synergistic effect with PD-L1 blockade (Ref. 67). C57, a PUS7 inhibitor, can effectively inhibit cell growth of GSC, and prolong the survival of mice with glioblastoma burden (Ref. 81) (Figure 2). Moreover, the demethylation of m1A by α-ketoglutarate-dependent dioxygenase human AlkB homolog 3 (ALKBH3) has been identified as crucial for the nucleosome formation of the promyelocytic leukemia (PML) protein, offering a novel therapeutic approach (Ref. 118). The METTL1/WDR4 complex, a key regulator of m7G modification, has been connected to various types of cancers and presents itself as a promising candidate for cancer therapy (Ref. 119). Also, the identification of altered patterns of rRNA pseudouridylation in BC suggests the potential use of pseudouridyl-modified rRNA sites for developing therapeutic strategies targeting BC (Ref. 120). Moreover, studies indicate that the high expression of YBX1 in OC cells can recognize the m5C modification on CHD3 mRNA, and YBX1 inhibitor SU056 can reverse the platinum resistance in animal models, suggesting that inhibition of YBX1 may be a potential strategy to overcome platinum resistance in OC (Ref. 121). Lastly, the miRNA mimics demonstrate significant potential in the clinical trials for cancer therapy; however, they encounter challenges related to stability and off-target effects. The new generation of molecular mimics enhances the stability of RNA oligonucleotides and minimizes the off-target effects, thereby facilitating their clinical application (Ref. 122).

Chemically modified ncRNAs present significant potential as therapeutic targets; however, several practical challenges hinder the development of therapies based on these modifications. One promising strategy is exosome-mediated delivery, which leverages the natural capacity of exosomes to transport miRNAs and evade phagocytosis. Nonetheless, issues such as the immunogenicity associated with allogeneic exosomes, along with challenges related to large-scale production and high manufacturing costs, must be addressed. Furthermore, the pharmacokinetic properties of ncRNAs pose limitations on their clinical application. Although chemical modifications can enhance RNA stability and bioavailability, further optimization of their metabolism and distribution *in vivo* is necessary (Ref. 123). Additionally, synthetic nanoparticles, including lipid nanoparticles, have demonstrated potential in the delivery of ncRNAs; however, their effectiveness in targeting specific cells while minimizing off-target effects remains a concern (Refs 124, 125). In terms of stability, chemical modifications, such as Nm modification, have proven effective in enhancing the stability of ncRNAs and reducing their immunogenicity (Ref. 122). Nevertheless, additional research is required to optimize these modifications and ensure their safety and efficacy in clinical applications (Ref. 123). Regarding potential side effects, the off-target effects of ncRNAs and their capacity to activate the immune system are significant issues (Ref. 126). Although advancements have been made in mitigating immunogenicity through chemical modifications, the long-term implications of these modifications on the immune system are not yet fully understood.

## Conclusions and future prospects

**Figure 3.**
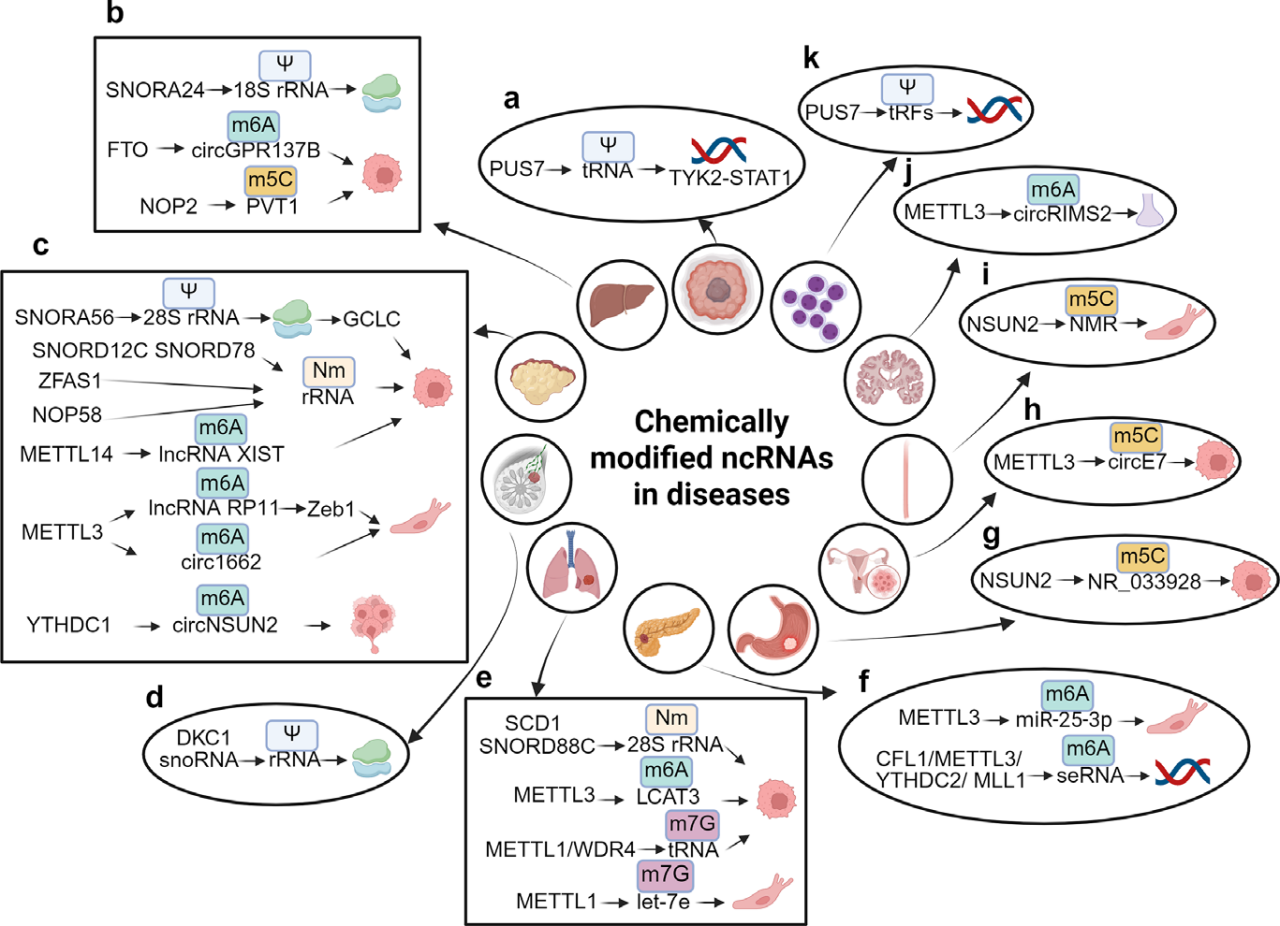
Chemically modified ncRNAs in diseases. (**A**) PUS7 facilitates the Nm modification of tRNA, which in turn regulates the TYK2-STAT1 signaling pathway in glioblastoma stem cell (GSC). (**B**) SNORD24 mediates the Ψ modification of 18S rRNA, affecting the structural functionality of ribosomes. FTO is responsible for the demethylation of m6A in circGPR137B, thereby inhibiting cell proliferation, while NOP2 mediates the m5C modification of PVT1, which promotes cell proliferation in hepatocellular carcinoma (HCC). (**C**) SNORA56 mediates the Ψ modification of 28S rRNA, thereby regulating the translation of the catalytic subunit of glutamate cysteine ligase (GCLC) and promoting cell proliferation. ZFAS1 recruits SNORD12C and SNORD78 through synergistic recruitment with NOP58, leading to the elevation of the Nm modification of rRNA and the promotion of cell proliferation. METTL14 downregulates m6A modification of lncRNA XIST, thereby facilitating cell proliferation, while METTL3 mediates m6A modification of lncRNA RP11 and circ1662, both of which enhance cell migration. Furthermore, YTHDC1 mediates m6A modification of circNSUN2, promoting liver metastasis of colorectal cancer (CRC). (**D**) snoRNA and DKC1 mediate the Ψ modification of rRNA, which affects ribosomal function in breast cancer (BC). (**E**) SNORD88C and SCD1 mediate the Nm modification of 28S rRNA, promoting cell proliferation in non-small cell lung cancer (NSCLC). METTL3 also mediates m6A modification of LCAT3, contributing cell proliferation, while METTL1 and the WD repeat domain 4 protein (WDR4) mediate m7G modification of tRNA, which promotes cell proliferation in lung adenocarcinoma (LUAD). Conversely, METTL1 mediates m7G modification of let-7e, inhibiting cell migration in lung cancer (LC). (**F**) METTL3 mediates m6A modification of miR-25-3p, promoting cell migration. The complex CFL1/METTL3/YTHDC2/MLL1 mediates m6A modification of super-enhancer RNA (seRNA), which promotes oncogene transcription in pancreatic ductal adenocarcinoma (PDAC). (**G**) NSUN2 mediates m5C modification of NR_033928, promoting cell growth in gastric cancer (GC). (h) METTL3 mediates m5C modification of circE7, inhibiting cell growth in cervical cancer (CC). (**I**) NSUN2 mediates m5C modification of NMR, promoting cell migration in esophageal squamous cell carcinoma (ESCC). (**J**) METTL3 mediates m6A modification of circRIMS2, which is implicated in synaptic and memory impairments associated with Alzheimer’s disease (AD). (**K**) PUS7 mediates the Ψ modification of tRFs to inhibit the synthesis of aberrant proteins, thereby improving hematopoietic function and protecting against leukemic progression.

Recent research has shown that ncRNAs are essential in regulating factors involved in chemical modifications through various mechanisms, while these factors, in turn, influence the biogenesis, stability, and functions of ncRNAs through site-specific modifications (Ref. 66). Researches on RNA modifications in ncRNAs have led to significant advancements, ranging from the identification of ncRNAs, the discovery of novel chemical modifications to advances in techniques for measuring chemical modifications (e.g., single-nucleotide-resolution mapping and nanopore sequencing) (Refs 6, 83, 127, 128). Each of these methods possesses distinct advantages and disadvantages, as well as varying sensitivity levels, which can result in inconsistent findings. For example, certain techniques may be unable to identify specific types of chemical modifications or may exhibit differing levels of accuracy in quantifying the extent of these modifications. Although ncRNA modifications are involved in other diseases (Refs 129, 130) (Figure 3), we focus on their regulatory patterns and clinical relevance in cancer. These modifications exhibit significant heterogeneity across cancer types, with distinct differences in function, distribution, and clinical significance (Refs 131, 132). The genetic background of various cancer types, such as FTO amplification and mutations in isocitrate dehydrogenase 1 (IDH1), along with the characteristics of the tumor microenvironment, including factors like hypoxia and immune cell infiltration, play a crucial role in influencing the expression and functionality of ncRNAs. This influence is mediated by the dynamic modulation of RNA chemical modifications, which subsequently contributes to tumor heterogeneity (Ref. 6). These discoveries have laid a solid foundation in biology and have underscored the significance of ncRNAs in cancer research.

Despite advancements in the field, several critical questions remain regarding the influence of ncRNA modifications on cancer. (1) It is essential to investigate how enzymes that facilitate chemical modifications select their RNA substrates and whether this selection is dependent on RNA sequence. (2) The dual roles of certain enzymes, such as METTL3, which may act as either oncogenes or tumor suppressors in cancer development (Ref. 133), require further clarification regarding the specific mechanisms that govern these opposing roles. (3) The potential interactions between different ncRNA modifications and their effects are necessary to be explored. (4) Given that alterations in the overall modification status can affect the role of numerous genes, there is an urgent need to develop more effective strategies for the detection of chemically modified ncRNAs. (5) The dynamic and low-abundance nature of RNA modifications present significant challenges to achieving quantitative accuracy, highlighting the necessity for standardized, high-throughput methodologies that can detect a wide array of chemically modified ncRNAs with spatiotemporal precision. (6) Discrepancies in research findings may arise from differences in experimental designs, methodologies, and models; for instance, variations in miRNA immunogenicity across studies complicate the prediction of immune responses and underscore the need for preclinical screening with human cells (Ref. 134). (7) The measurement of ncRNA modifications is technically challenging, requiring precise methods to detect and quantify specific chemical alterations. To enhance the accuracy of ncRNA modification profiling, several potential solutions have been proposed. Recent studies suggest that the integration of multiple high-throughput sequencing techniques could significantly improve the precision of ncRNA modification analysis. Additionally, the development of more sensitive and specific antibodies targeting modified ncRNAs may further enhance the accuracy of both detection and quantification. (8) Off-target effects represent a considerable concern, often resulting from sequence similarities or excessive dosing, which can lead to unintended interactions with non-target RNAs (Ref. 135). On the one hand, off-target gene silencing can result from unintended binding, leading to inadvertent silencing or activation of genes, which may obscure experimental outcomes. On the other hand, extensive off-target activity has the potential to disrupt critical genes or non-coding regions, potentially inducing apoptosis or causing genomic instability. To mitigate these challenges, several strategies may be employed. Firstly, the incorporation of locking nucleic acid (LNA) and unlocked nucleic acid (UNA) modifications can enhance the specificity of guide strand selection, thereby reducing the likelihood of off-target effects. Secondly, the development of efficient targeted delivery systems can facilitate the precise delivery of therapeutic agents to intended cells or tissues, thereby minimizing their distribution and impact on non-target sites. Lastly, the application of high-throughput sequencing and other advanced technologies can enable a comprehensive evaluation of potential off-target effects associated with therapeutic agents, allowing for the timely exclusion of drug candidates that pose significant off-target risks and ensuring that only those with high safety and efficacy profiles progress to clinical trials. (9) Challenges related to delivery, such as achieving efficient and specific targeting of cells while minimizing effects on non-target cells and avoiding activation of the innate immune system, further hinder the clinical translation of these findings.

In conclusion, exploring ncRNA modifications in cancer is an emerging research domain, offering the possibility to uncover a multitude of differentially expressed ncRNAs that could serve as early diagnostic biomarkers. This work not only aims to reveal the interaction mechanisms underlying between chemically modified ncRNAs and cancer development but also holds promise for the creation of innovative cancer therapies. Future research endeavors should focus on the establishment of standardized methodologies for the quantification of chemical modifications, thereby facilitating the comparability of data across various studies. Additionally, longitudinal studies are essential to evaluate the persistence and safety of chemically modified ncRNAs in the context of cancer treatment. Furthermore, the implementation of clinical trials is imperative to assess the efficacy of these ncRNAs and to compile data pertinent to their medical application. Pursuing these research trajectories will contribute to the transition of ncRNA therapies from the laboratory to the clinic, with the potential to improve cancer treatment outcomes.
